# Inhibition of thioredoxin activates mitophagy and overcomes adaptive bortezomib resistance in multiple myeloma

**DOI:** 10.1186/s13045-018-0575-7

**Published:** 2018-02-27

**Authors:** Zhihong Zheng, Shengjun Fan, Jing Zheng, Wei Huang, Cristina Gasparetto, Nelson J. Chao, Jianda Hu, Yubin Kang

**Affiliations:** 1 Department of Hematology, Fujian Provincial Key Laboratory of Hematology, Fujian Medical University Union Hospital, 29 Xinquan Road, Fuzhou, Fujian 350001 China; 2 Division of Hematologic Malignancies and Cellular Therapy, Duke University Medical Center, 3961, Durham, NC 27710 USA

**Keywords:** Multiple myeloma, Bortezomib, Resistance, Thioredoxin, Mitophagy

## Abstract

**Background:**

Although current chemotherapy using bortezomib (Velcade) against multiple myeloma in adults has achieved significant responses and even remission, a majority of patients will develop acquired resistance to bortezomib. Increased thioredoxin level has been reported to be associated with carcinogenesis; however, the role of thioredoxin in bortezomib drug resistance of myeloma remains unclear.

**Methods:**

We generated several bortezomib-resistant myeloma cell lines by serially passaging with increased concentrations of bortezomib over a period of 1.5 years. Thioredoxin expression was measured by real-time PCR and western blot.

**Results:**

The role of thioredoxin in the survival of bortezomib-resistant myeloma cells was determined by specific shRNA knockdown in vitro and in vivo. Thioredoxin inhibitor (PX12) was used to determine the effectiveness of thioredoxin inhibition in the treatment of bortezomib-resistant myeloma cells. The effect of thioredoxin inhibition on mitophagy induction was examined. The correlation of thioredoxin expression with patient overall survival was interrogated. Thioredoxin expression was significantly upregulated in bortezomib-resistant myeloma cells and the change correlated with the increase of bortezomib concentration. Thioredoxin gene knockdown using specific shRNA sensitized bortezomib-resistant myeloma cells to bortezomib efficiency in vitro and in vivo. Similarly, pharmacological inhibition with PX12 inhibited the growth of bortezomib-resistant myeloma cells and overcame bortezomib resistance in vitro and in vivo. Furthermore, inhibition of thioredoxin resulted in the activation of mitophagy and blockage of mitophagy prevented the effects of PX12 on bortezomib-resistant myeloma cells, indicating that mitophagy is the important molecular mechanism for the induction of cell death in bortezomib-resistant myeloma cells by PX12. Moreover, inhibition of thioredoxin resulted in downregulation of phosphorylated mTOR and ERK1/2. Finally, thioredoxin was overexpressed in primary myeloma cells isolated from bortezomib-resistant myeloma patients and overexpression of thioredoxin correlated with poor overall survival in patients with multiple myeloma.

**Conclusions:**

Our findings demonstrated that increased thioredoxin plays a critical role in bortezomib resistance in multiple myeloma through mitophagy inactivation and increased mTOR and ERK1/2 phosphorylation. Thioredoxin provides a potential target for clinical therapeutics against multiple myeloma, particularly for bortezomib-resistant/refractory myeloma patients.

**Electronic supplementary material:**

The online version of this article (10.1186/s13045-018-0575-7) contains supplementary material, which is available to authorized users.

## Background

Multiple myeloma, also referred as plasma cell myeloma, plasmacytic myeloma, myelomatosis, or Kahler disease, is a neoplastic malignancy characterized by the proliferation of abnormal plasma cells derived from B cells [1, 2]. These plasma cells proliferate in the bone marrow and frequently invade into the adjacent bone, causing skeletal destruction that finally results in fractures [3, 4]. In addition, multiple myeloma can cause anemia, renal insufficiency, frequent infection, and hypercalcemia and is associated with significant mortality and morbidity. Multiple myeloma accounts for about 1% of all types of malignancy and 10% of hematologic malignancies. In 2017, there were 30,280 new diagnoses and an estimated 12,590 deaths from myeloma in the USA alone. Existing therapies for multiple myeloma, including the proteasome inhibitors bortezomib (BTZ) and carfilzomib, have the potential of extending the overall survival but are not curative [5]. Bortezomib is one of the major drugs in the treatment of multiple myeloma [6]. Almost all myeloma patients will receive bortezomib during their course of treatment, yet nearly all patients will develop drug resistance to bortezomib [7]. Thus, to unmask the failure to cure, drug resistance mechanisms associated with bortezomib must be characterized.

Thioredoxin (TXN) is a 12 kDa ubiquitous oxidoreductase. Along with NADPH and thioredoxin reductase, thioredoxin constitutes the thioredoxin system, one of the major disulfide reductase antioxidant systems in mammalian cells. Thioredoxin is crucial in defense against oxidative stress and in maintaining the redox environment in the cell. In addition, TXN has multi-faceted roles [8]. TXN regulates a variety of redox-sensitive signaling pathways as well as ROS-independent genes and exerts cytoprotective effects. Overexpression of thioredoxin has been implicated in the pathogenesis of advanced malignancies, including solid cancer and hematological malignancy [9]. Besides, there are increasing evidences of its role in the development of resistance to several chemotherapeutic agents including cisplatin and docetaxel [10, 11].

Mitophagy, mitochondrial degradation by autophagic delivery to lysosomes, is the major degradative pathway in mitochondrial turnover. Mitochondria are the essential site of aerobic energy production in eukaryotic cells. Maintaining a healthy population of mitochondria is essential to the cellular redox homeostasis and the well-being of cell self-renewal [12]. The role of mitophagy dysfunction in cancer pathogenesis is currently an area of active investigation but the findings varied [13]. For instance, some studies suggested that a decrease in mitophagy led to the increase in the production of free radicals and subsequent genetic instability [14, 15], thus, favoring the development of cancer. On the other hand, studies have found that mitophagy protected cancer cells from apoptosis [16, 17]; thus, an increase in mitophagy will promote cancer cell survival and progression. It is highly likely that the double-edged roles of mitophagy dysfunction in cancer pathogenesis may change significantly depending on cancer cell types [18]. However, the role of mitophagy in chemotherapeutics-induced drug resistance is still unknown and remains to be investigated.

We hypothesized that thioredoxin plays an important role in bortezomib drug resistance in multiple myeloma, providing a novel therapeutic target against cancer drug resistance in the treatment of multiple myeloma. Additionally, we aimed at investigating the effects of thioredoxin on mitophagy in bortezomib-resistant myeloma cells.

## Methods

### Cell culture and generation of bortezomib-resistant myeloma cells

The human multiple myeloma cell lines MM.1S, MM.1R, OPM1, RPMI8226/Dox, and NCIH929 were purchased from ATCC Company. Cells were grown in suspension in RMPI1640 medium supplemented with 10% fetal bovine serum, 1% (*v*/*v*) penicillin, and 100 μg/mL streptomycin. Cells were maintained at 37 °C in a 5% CO_2_ atmosphere with a proper humidity.

To generate bortezomib-resistant myeloma cell lines, bortezomib (BTZ) was added to the multiple myeloma cell culture medium starting at 0.03 nM. The culture medium was replaced with bortezomib containing medium twice weekly and the myeloma cells were cultured for 2–4 weeks until the cells survived and became resistant to that concentration of bortezomib. The bortezomib concentration was then increased by doubling the previous concentration until BTZ reached at a final concentration of up to 8.4 nM by the end of the second year. Myeloma cell lines were maintained on BTZ containing medium until 1 week before experiments.

### Drugs and reagents

The proteasome inhibitor bortezomib (PS-341, Velcade®) was obtained from Selleckchem Chemicals LLC (Houston, TX). The thioredoxin inhibitor 2-[(1-methylpropyl) dithio]-1H-imidazole (PX12) was purchased from Tocris Bioscience (Bristol, UK). The mitophagy inhibitor bafilomycin was obtained from Sigma-Aldrich (St. Louis, Missouri, USA). 3-(4,5-dimethylthiazol-2-yl)-2,5-diphenyltetrazoliumbromide (MTT) were purchased from GIBCO BRL (Grand Island, NY). The mTOR activator (MHY1485) and ERK activator (tert-butylhydroquinone; tBHQ) were purchased from Sigma-Aldrich (St. Louis, Missouri, USA). Drugs were dissolved in dimethyl sulfoxide (DMSO; Sigma-Aldrich, St. Louis, Missouri, USA) at a concentration of 100 mM as a stock solution. Anti-PINK1 antibody (ab75487) and anti-LC3B antibody (ab51520) used for western blot analysis were purchased from Abcam (Cambridge, MA). Anti-beta-actin antibody (A2228) was obtained from Sigma-Aldrich. Anti-thioredoxin antibody (C63C6), anti-AKT antibody (9272), anti-mTOR antibody (2972), anti-phospho-Akt (Ser473, 4060) antibody, anti-phospho-mTOR (Ser2448, 5536) antibody, and anti-phospho-p44/42 MAPK (ERK1/2, Thr202/Tyr204, 9101) antibody were purchased from Cell Signaling Technology Inc. (Beverly, MA, USA). Human CD138 enrichment kit (EasySep™) was purchased from StemCell Technologies (Vancouver, BC, Canada).

### MTT assay

Equal number of parental and BTZ-resistant multiple myeloma cells were seeded in 96-well plates. Different concentrations of BTZ with or without PX12 were added to the cells in each group (at least four replicates for each group). Cells were incubated in RPMI1640 medium at 37 °C in a 5% CO_2_ humidified atmosphere. After incubating for 48 or 72 h, 10 μL MTT (5 mg/mL) was added to each well and plates were incubated at 37 °C for another 4 h. Finally, 100 μL of 10% sodium dodecyl sulfate (with 0.01 N HCl) was added to dissolve the crystals and absorbance was determined at 570 nm in an EL340 microplate reader (BioTek Instruments, Winooski, VT). Ratios of the 50% inhibitory concentration (IC_50_) value in BTZ-resistant groups to the IC_50_ value of parental cells were calculated and considered to be the relative indicators of drug resistance in the experimental groups.

### Gene expression analyses

For *TXN* mRNA expression (forward: 5′-GTAGTTGACTTCTCAGCCACGTG-3′, reverse: 5′-CTGACAGTCATCCACATCTACTTC-3′), total RNAs were extracted using TRIzol reagent (Invitrogen) according to standard procedures and reverse transcribed into complementary DNA (cDNA) using a BIO-RAD iScript ™ cDNA synthesis kit (Bio-Rad, Hercules, CA, USA). Samples were then analyzed using an Applied Biosystems Real-Time PCR (SYBR Green, Bio-Rad Laboratories, Hercules, CA, USA) in triplicate. Gene expression was normalized using 18S rRNA (forward: 5′-GTAACCCGTTGAACCCCATT-3′, reverse: 5′-CCATCCAATCGGTAGTAGCG-3′).

### Western blot analysis

Western blot analysis was performed as previously described [19]. Briefly, total protein was extracted using a RIPA buffer (50 mM Tris HCl, pH 7.4/150 mM NaCl/5 mM EDTA/1% NP-40/1% sodium deoxycholate/0.1% SDS/1% aprotinin, 50 mM NaF/0.1 mM Na_3_VO_4_), and equal amounts of proteins were separated using a SDS-PAGE electrophoresis. Separated proteins were then transferred to polyvinylidene difluoride membranes (PVDF; Millipore Corp., Bedford, MA, USA) and incubated with primary antibodies for thioredoxin (1:1000), PINK1 (1:200), LC3B (1:1000), AKT/pAKT (1:1000), mTOR/p-mTOR (1:1000), pERK1/2 (1:1000), or β-actin (1:10,000) overnight at 4 °C with gentle agitation. Membranes were washed and then incubated with HRP-conjugated secondary antibodies (1:10,000) for 2 h at room temperature before signal detection by chemiluminescence (Pierce Biotechnology, Rockford, IL, USA). Densitometric quantification was performed by Image-Pro Plus 6.0 software (Media Cybernetics, Silver Springs, MD 20910, USA).

### Thioredoxin-specific shRNA knockdown

Plasmids targeting human thioredoxin (shTXN1-4, catalog number RHS4430-200171579, RHS4430-200174379, RHS4430-200273352, and RHS4430-200274993) were purchased from GE Healthcare (Piscataway, NJ, USA). Plasmid for non-targeting control (shNT) and the packing and envelope vectors psPAX2 and VSVG were obtained from Addgene (Cambridge, Massachusetts). HEK293T cells were transfected with shNT or shTXN, psPAX2, and VSV-G using Lipofectamine 2000 (Invitrogen, Carlsbad, CA, USA) for 24 h. The DMEM medium was changed and collected at 24 and 48 h after transfection, respectively. To obtain thioredoxin stably knockdown cells, the transduced cells were cultured with 1 μg/ml puromycin and the GFP^+^ cells were sorted and expanded.

### Mitochondrial network by transmission electron microscopy

Conventional transmission electron microscopy analysis was performed as described previously [20]. Briefly, human multiple myeloma cells with or without treatment were fixed by a solution containing 4% formaldehyde and 2% glutaraldehyde. Specimens were washed following by OSO_4_ postfixed, alcohol dehydrated, and araldite embedded. Thin sections of samples were analyzed using a FEI Tecnai G^2^ Twin electron microscope (FEI, Hillsboro, OR, USA).

### Determination of mitochondrial membrane potential (Δψm)

JC-1 fluorescent probe kit (Molecular Probes, Eugene, OR, USA) was used to determine Δψm with two different staining spectra, the orange aggregates dye form for normally respiring cells and green monomers for cells with respiratory dysfunction (apoptotic cells). Briefly, cells with or without treatment were incubated in RPMI1640 media containing JC-1 (2 μM final concentration) at 37 °C for 15 min. Cell pellets were resuspended in cold PBS and analyzed on a flow cytometer with 488-nm excitation emission.

### In vivo tumor xenograft model

All animal experiments were approved by the Animal Care Committee of Duke University Medical Center. NOD/LtSz-scid/scid (NOD/SCID) mice were purchased from Jackson Laboratories (Bar Harbor, ME, USA) and maintained in microisolator cages on laminar flow racks under pathogen-free conditions in the Division of Laboratory Animal Resources, Duke University. BTZ-resistant MM.1R multiple myeloma cells, 3 × 10^6^ cells in 100 μL PBS, were injected subcutaneously into the dorsal lateral flank of NOD/SCID mice that had received a total body irradiation with 1.5 Gy from a ^137^Cs source. Engraftment of human myeloma was monitored twice per week. When the tumor was established and palpable after 10 days of xenograft, the mice were given control buffer, PX12 (12 mg/kg, i.p., twice weekly), bortezomib (0.5 mg/kg, i.p., twice weekly) or the combination of PX12, and bortezomib. Tumor length and width were measured with a caliper and tumor volume was calculated using the formula *V* = (*L* × *W* × *W*)/2, where *V* is the tumor volume, *L* is the tumor length, and *W* is the tumor width. At the end of the experiments, tumors were harvested and weighed. A portion of the tumors were homogenized and used for western blot analysis.

### Microarray data mining

For oncomining gene expression analysis, mRNA level of *TXN* in normal plasma cell and myeloma cells was queried using Oncomine database (https://www.oncomine.org). The prognostic value of *TXN* in multiple myeloma was assessed using Mulligan and Arkansas myeloma microarrays. Overall survival was compared between high and low expression of *TXN* using median gene expression value as a bifurcating point.

### Patient samples

Bone marrow aspirates were obtained from patients with myeloma with patients’ informed consent and IRB approval. The study was performed in compliance with the guidelines of the Ethical Committee of Duke University Medical Center. A total of 13 patients with myeloma, including three newly diagnosed myeloma, five relapsed myeloma, and five treated myeloma prior to stem cell transplant were enrolled in the study. Human CD138^+^ and CD138^−^ cells were isolated from the bone marrow aspirates of these patients using Histopaque-1077 (Sigma) gradient separation followed by human CD138 enrichment kit (EasySep™, StemCell Technologies). The purity of the human CD138^+^ cells was > 95%.

### Statistical analyses

All statistical analyses were performed using the Student’s *t* test and represented as mean ± standard error of the mean (SEM) unless noted otherwise. For in vivo experiments with ≥ 5 per group, statistical analyses were performed with two-way ANOVA followed by multiple comparisons. The *p* values were designated as *p < 0.05, **p < 0.01, ***p < 0.005, ****p < 0.001; *n.s.* non-significant (*p* > 0.05).

## Results

### Upregulation of thioredoxin correlates with the development of bortezomib drug resistance in multiple myeloma cells

**Fig. 1:**
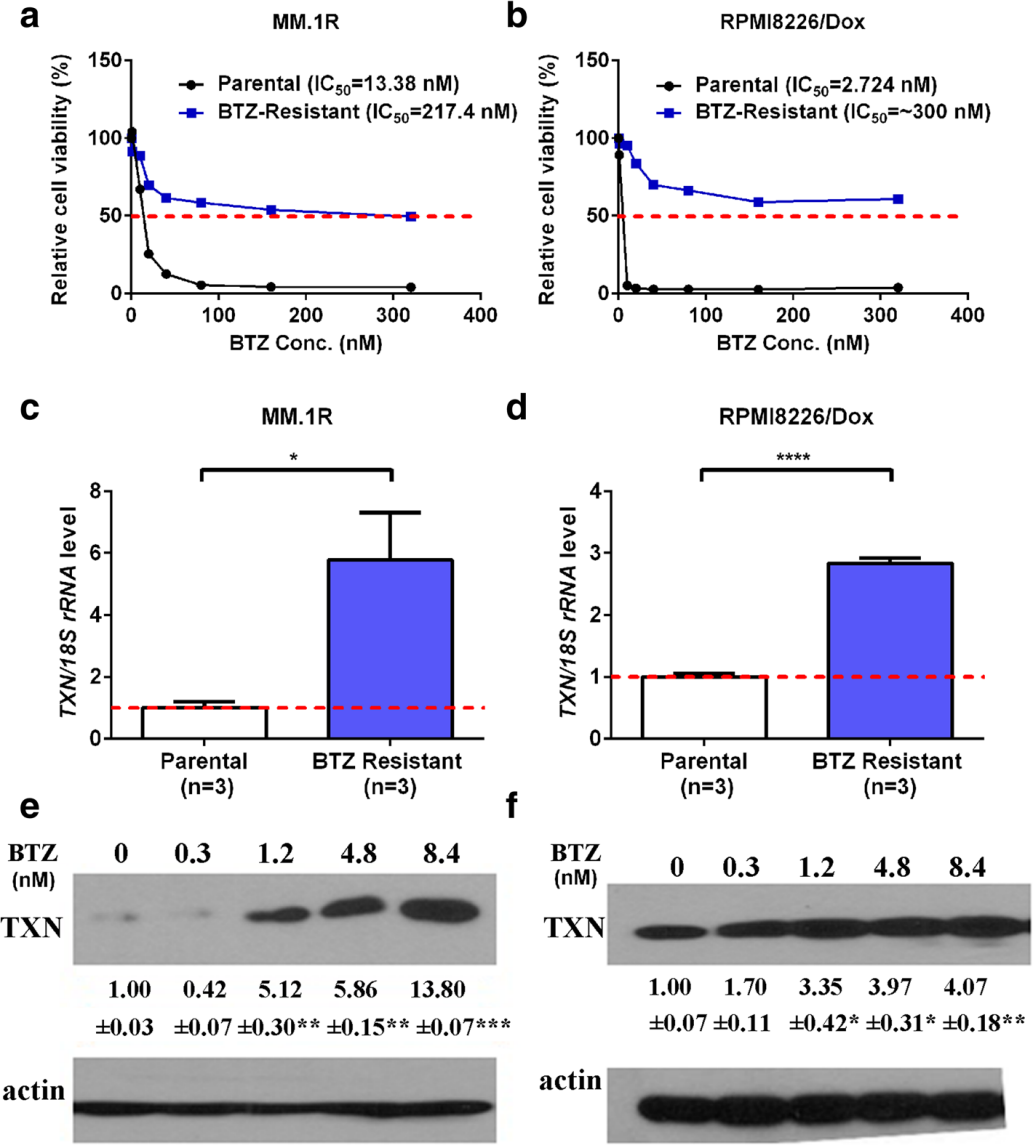
Thioredoxin is upregulated in adaptive bortezomib-resistant multiple myeloma cells. **a** and **b** Generation of adaptive bortezomib-resistant myeloma cell lines**:** MM.1R cells (**a**) and RPMI8226/Dox cells (**b**) were cultured with serially increased concentrations of bortezomib over a period of 1.5 years. The parental and bortezomib-resistant myeloma cells were treated with various concentration of bortezomib for 48 h and the IC_50_ for parental and bortezomib-resistant myeloma cells were calculated and shown. **c** and **d** Thioredoxin mRNA expression is upregulated in bortezomib (BTZ)-resistant MM.1R cells (**c**) and in RPMI8226/Dox cells (**d**). Parental cells and BTZ-resistant MM.1R cells and BTZ-resistant RPMI8226/Dox cells at the highest bortezomib concentration were harvested and mRNA expression measured by RT-PCR. Error bars, standard error of the mean (SEM) (**e** and **f**). Thioredoxin protein expression increased in parallel to increased bortezomib drug resistance. Serial MM.1R (**e**) and RPMI8226/Dox (**f**) cells that were cultured with increased concentrations of bortezomib were harvested and protein expression measured by western blot analysis. The intensity of expression was semi-quantitated using Image-Pro Plus 6.0 software and adjusted to β-actin. Error bars, standard error of the mean (SEM); *p < 0.05, **p < 0.01, ***p < 0.005, ****p < 0.001

To generate adaptive BTZ-resistant multiple myeloma cell lines, we cultured five different myeloma cell lines (MM.1S, MM.1R, OPM1, RPMI8226/Dox, and NCIH929) over a period of 1.5 years with gradually increased concentrations of BTZ. At the end of the serial cultures, the IC_50_ of the BTZ-resistant myeloma cell lines to BZT has increased by 10–100 fold (ranging from 7.06 to 110 fold) (Fig. 1a, b and Additional file 1: Fig. S1A–1C). Compared to their parental BTZ-sensitive cells, BTZ-resistant myeloma cells demonstrated a significant upregulation of both thioredoxin mRNA gene expression and protein expression (Fig. 1c–f and Additional file 1: Fig. S1D–1I). Importantly, thioredoxin protein expression increased in parallel to the increase in drug resistance to BTZ (Fig. 1e, f). These data suggested that thioredoxin plays an important role in the development of bortezomib drug resistance in multiple myeloma.

### Genetic knockdown of thioredoxin sensitizes bortezomib-resistant myeloma cells to bortezomib

**Fig. 2:**
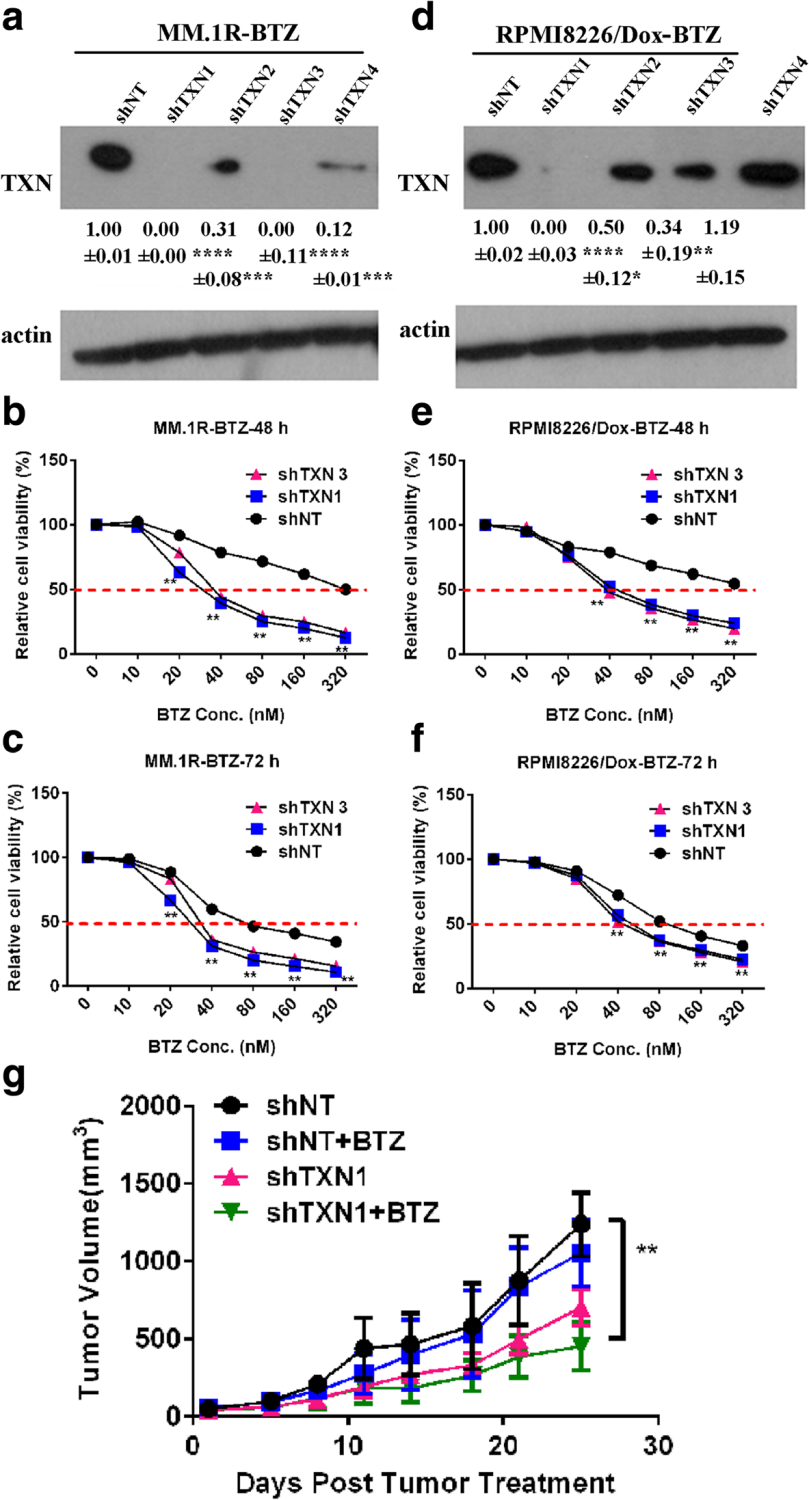
Genetic deletion of thioredoxin reduced BTZ-resistant multiple myeloma growth both in vitro and in vivo in response to bortezomib treatment. **a** Knockdown efficiency of thioredoxin-specific shRNAs in MM.1R-BTZ-resistant cells 48 h after transfection. **b** Cell viability of MM.1R-BTZ-resistant cells after transfecting with shNT, shTXN1, and shTXN3 for 48 h. **c** Cell viability of MM.1R-BTZ-resistant cells after transfecting with shNT, shTXN1, and ShTXN3 for 72 h. **d** Knockdown efficiency of thioredoxin-specific shRNAs in RPMI8226/Dox-BTZ-resistant cells 48 h after transfection. **e** Cell viability of RPMI8226/Dox-BTZ-resistant cells after transfecting with shNT, shTXN1, and ShTXN3 for 48 h. **f** Cell viability of RPMI8226/Dox-BTZ-resistant cells after transfecting with shNT, shTXN1, and ShTXN3 for 72 h. **g** Tumor volume in mice xenografted with MM.1R-BTZ-resistant cells transduced with shNT or shTXN1. MM.1R-BTZ-resistant cells transduced with shNT or shTXN1 were injected subcutaneously into 1.5 Gy-irradiated NOD/SCID mice. Ten days later, the mice were treated with buffer control or bortezomib at 0.5 mg/kg, i.p., twice weekly. The intensity of expression was semi-quantitated using Image-Pro Plus 6.0 software and adjusted to β-actin. Error bars, standard error of the mean (SEM); *p < 0.05, **p < 0.01, ***p < 0.005, ****p < 0.001

We next determined the role of thioredoxin in bortezomib drug resistance using shRNA-specific gene knockdown techniques. Four shRNAs specific for human thioredoxin were transfected into MM.1R-BTZ-resistant myeloma cells and RPMI8226/Dox-BTZ-resistant myeloma cells. Thioredoxin knockdown efficiencies were determined by western blot. As shown in Fig. 2a, d, shTXN1 and shTXN3 were very effective in downregulating thioredoxin expression in both MM.1R-BTZ and RPMI8226/Dox- BTZ cells and were chosen for subsequent experiments.

MM.1R-BTZ cells transduced with shTXN1 or shTXN3 showed increased sensitivity to bortezomib after 48 and 72 h of incubation in comparison to MM.1R-BTZ cells transduced with mock control shNT (Fig. 2b, c). A similar phenotype was observed in RPMI8226/Dox-BTZ cells transduced with shTXN1 or shTXN3 (Fig. 2e, f). We further explored the effects of thioredoxin gene knockdown on overcoming bortezomib resistance in vivo. MM.1R-BTZ cells transduced with shNT and MM.1R-BTZ cells transduced with shTXN1 were subcutaneously injected into 1.5 Gy total body irradiated NOD/SCID mice. Ten days later when the tumor was established, the mice were then treated with or without bortezomib (0.5 mg/kg, i.p., twice weekly). Tumor growth was measured. MM.1R-BTZ cells transduced with shNT formed tumor mass at day 10 post inoculation and progressed rapidly up to 1100 mm^3^ at day 25 after xenograft into the immunodeficient mice. Treatment with bortezomib did not affect tumor growth in MM.1R-BTZ cells transduced with shNT. On the other hand, MM.1R-BTZ cells transduced with shTXN responded to bortezomib treatment and the tumor sizes of MM.1R-BTZ-shTXN1 cells were significantly smaller in size (451.75 ± 38.82 mm^3^ at day 25) when compared with MM.1R-BTZ-shNT tumors (Fig. 2g, *p* = 0.0003). These data suggested that genetic deletion of thioredoxin could abolish the cell growth advantage in BTZ-resistant multiple myeloma cells and re-sensitize these cells to bortezomib treatment.

### Treatment with thioredoxin inhibitor (PX12) inhibits the growth of bortezomib-resistant myeloma cells and re-sensitizes bortezomib-resistant myeloma cells to bortezomib

**Fig. 3:**
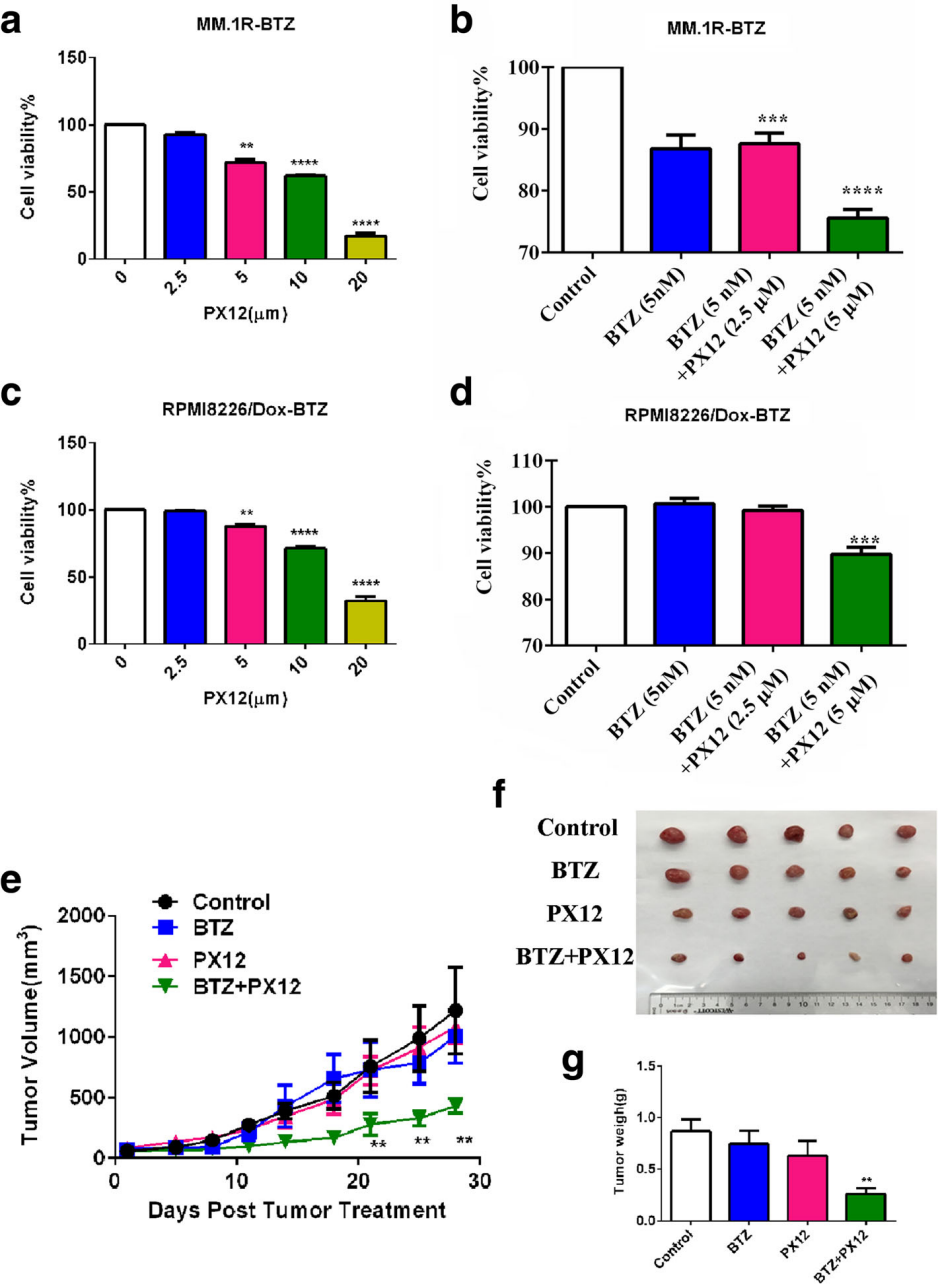
Pharmacological inhibition of thioredoxin confers BTZ-resistant myeloma cells growth arrest both in vitro and in vivo. **a** Cell viability of MM.1R-BTZ-resistant cells following 48 h of PX12 treatment. **b** Effect of PX12 on MM.1R-BTZ-resistant cells following 48 h of BTZ co-treatment. **c** Cell viability of RPMI8226/Dox-BTZ-resistant cells following 48 h of PX12 treatment. **d** Effect of PX12 on RPMI8226/Dox-BTZ-resistant cells following 48 h of BTZ co-treatment. **e** Tumor growth curves of the subcutaneous xenograft tumor model with MM.1R-cells treated with control buffer, BTZ alone, PX12 alone or the combination of BTZ and PX12. **f** Representative images of MM.1R-BTZ-resistant tumor dissected from sacrificed mice are shown in this panel. **g** Tumor weight in mice xenograft with MM.1R-BTZ-resistant cells treated with control buffer, BTZ alone, PX12 alone, or the combination of BTZ and PX12. For this experiment, mice were treated with either BTZ (0.5 mg/kg, twice weekly, i.p.), PX12 (12 mg/kg, twice weekly, i.p.) or the combination. Error bars, standard error of the mean (SEM); *p < 0.05, **p < 0.01, ***p < 0.005, ****p < 0.001

We tested the effects of thioredoxin inhibitor on the survival and drug resistance in bortezomib-resistant myeloma cells in vivo and in vitro. PX12, a commercially available thioredoxin-specific inhibitor, binds to the Cys73 residue of thioredoxin, causing it to become irreversibly thioalkylated and biologically inactive [21]. We found that PX12 inhibited the growth of MM.1R-BTZ and RPMI8226/Dox-BTZ cells in a dose-dependent manner **(**Fig. 3a, c). When PX12 was combined with bortezomib, it could re-sensitize bortezomib-resistant myeloma cells to bortezomib (Fig. 3b, d). We next explored the pharmacological therapeutic potential of PX12 for the treatment of BTZ-resistant multiple myeloma in vivo. For this purpose, equal volumes of MM.1R-BTZ tumor cells were xenografted into the NOD/SCID mice. Two weeks later when the tumor was formed, mice were treated with control buffer, BTZ alone (0.5 mg/kg, twice per week injection, i.p.), PX12 (12 mg/kg, twice per week, i.p.), or the combination for additional 4 weeks. As shown in Fig. 3e–g, the combination of PX12 with BTZ treatment had marked effects against MM.1R-BTZ tumor growth in NOD/SCID mice. Taken together, our findings demonstrate the potential of targeting thioredoxin to re-sensitize BTZ-resistant multiple myeloma to bortezomib treatment.

### Inhibition of thioredoxin induces mitophagy in bortezomib-resistant myeloma cells

Mitophagy, the autophagy of mitochondria, is a highly specific quality control process and is currently under extensive investigation for its role in carcinogenesis, cancer progression, and resistance to chemotherapy [18]. Thioredoxin is an antioxidant and is important in maintaining redox homeostasis. We reasoned that inhibition of thioredoxin would affect mitochondrial function and lead to the activation of mitophagy. We further hypothesized that PX12 inhibited the growth of BTZ-resistant myeloma cells and re-sensitized BTZ-resistant myeloma cells to bortezomib through the activation of mitophagy. To test this hypothesis, we measured several mitophagy activation markers, including the conversion from LC3BI to LC3BII, the expression of PETN-induced putative kinase 1 (PINK1), and mitochondria membrane potential (Δψm by JC-1) as well as the fusion of mitochondria and autophagosome under transmission electron microscopy.

**Fig. 4:**
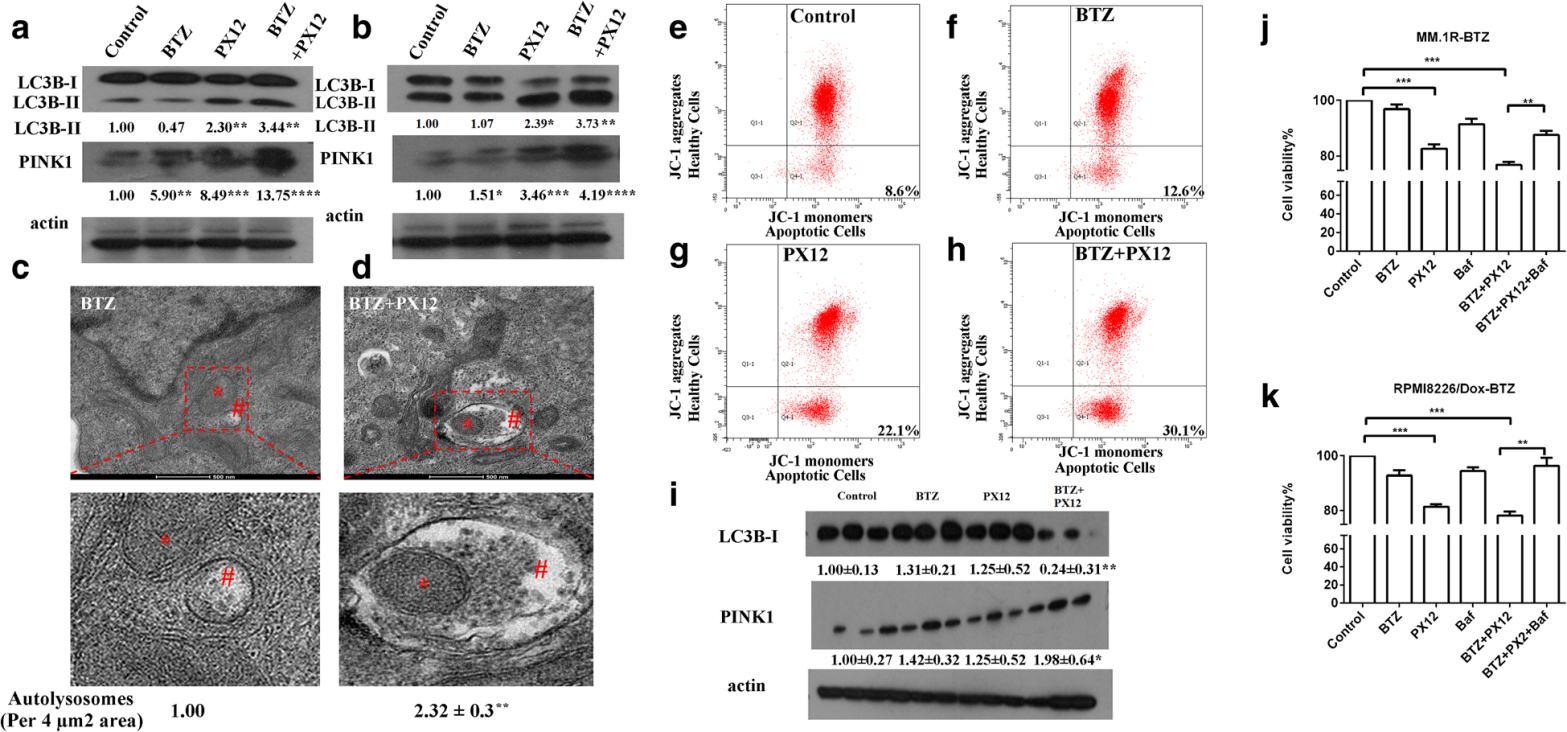
Pharmacological inhibition of thioredoxin by PX12 triggers mitophagy in BTZ-resistant multiple myeloma cells. **a** Western blot analyses of LC3B and PINK1 expression in MM.1R-BTZ-resistant cells treated with BTZ (5 nM), PX12 (2.5 μM), or BTZ+PX12 after 48 h of incubation. **b** Western blot analyses of LC3B and PINK1 expression in RPMI8226/Dox-BTZ-resistant cells treated with BTZ (5 nM), PX12 (2.5 μM), or BTZ+PX12 after 48 h of incubation. **c** Representative transmission electron microscopy images of mitochondria network in MM.1R-BTZ-resistant cells treated with BTZ (5 nM) for 48 h. **d** Representative transmission electron microscopy images of mitochondria network in MM.1R-BTZ-resistant cells treated with BTZ (5 nM) and PX12 (2.5 μM) combination for 48 h. Asterisk indicates mitochondria, while number sign indicates lysosomes. Scale bar: 500 nm. **e**–**h** The JC-1 fluorescence intensity in MM.1R-BTZ-resistant cells treated with BTZ (5 nM), PX12 (2.5 μM), or BTZ+PX12 were analyzed using flow cytometer. **i** Western blot analyses of LC3B and PINK1 expression in BTZ-resistant MM.1R tumor dissected from sacrificed mice treated with BTZ (0.5 mg/kg), PX12 (12 mg/kg), or the combination. **j** Cell viability of MM.1R-BTZ-resistant cells treated with BTZ (5 nM) or/and PX12 (2.5 μM) with or without bafilomycin (0.5 nM). **k** Cell viability of RPMI8226/Dox-BTZ-resistant cells treated with BTZ (5 nM) or/and PX12 (2.5 μM) with or without bafilomycin (0.5 nM). The intensity of expression was semi-quantitated using Image-Pro Plus 6.0 software and adjusted to β-actin. Error bars, standard error of the mean (SEM); *p < 0.05, **p < 0.01, ***p < 0.005, ****p < 0.001

Microtubule-associated protein 1A/1B-light chain 3 (LC3) is a soluble protein that is distributed ubiquitously in cells. During autophagy, the cytosolic form of LC3 (LC3-I) is conjugated to phosphatidylethanolamine to form LC3-phosphatidylethanolamine conjugate (LC3-II). Increase in LC3-II and/or reduction in LC3-I has become a reliable marker for autophagy and autophagy-related process [22]. MM.1R-BTZ cells and RPMI8226/Dox-BTZ cells were treated with bortezomib, PX12, or the combination of bortezomib and PX12, and LC3B-I and LC3B-II were measured by western blot analysis. As shown in Fig. 4a, b, PX12 treatment significantly increased LC3B-II expression compared to that in control group. The combination of PX12 and bortezomib had further increased LC3B-II expression in MM.1R-BTZ cells and RPMI8226/Dox-BTZ cells. PINK1 is a mitochondrial serine threonine kinase and serves as a mitophagy-specific marker [23]. Similar to LC3B-II, PX12 treatment increased PINK1 expression and the combination of PX12, and bortezomib had augmented effect on PINK1 expression in both MM.1R-BTZ- and RPMI8226/Dox-BTZ-resistant cells (Fig. 4a, b), indicating the activation of mitophagy.

To further elucidate the role of PX12 toward autophagic response in BTZ-resistant multiple myeloma, we applied transmission electron microscopy to visualize the mitophagy process and quantitated autolysosomes per 4 μm^2^ area. As shown in Fig. 4c, there were fewer intracellular autophagic vacuoles in MM.1R-BTZ cells treated with bortezomib. The mitochondrial was intact and separated from autophagosome. On the other hand, there was a significant increase (by 2.3 fold) in the autophagic response in MM.1R-BTZ cells treated with the combination of PX12 and BTZ, reflected by an increase in the amount of autophagy and lysosomes and the engulfed mitochondria by autophagosome (Fig. 4d). Additionally, we measured mitochondrial membrane potential (Δψm) using MitoProbe staining in MM.1R-BTZ cells treated with BTZ alone, PX12, or the combination. When mitochondrial membrane depolarizes (i.e., the loss of Δψm), JC-1 aggregates accumulate as demonstrated by the increase in the JC-1 fluorescence intensity in Q4 area (Fig. 4e–h). PX12 treatment as well as the combination of PX12 and BTZ led to accelerated Δψm loss. The percentage of MM.1R-BTZ cells undergoing mitophagy (the cells in Q4) drastically increase from 12.6% in bortezomib alone treatment group to 30.1% in bortezomib+PX12 combination group (Fig. 4f, h).

To determine if the combination of PX12 and bortezomib induced mitophay in vivo in tumor xenograft model, the tumors as described in Fig. 3 were harvested at the end of experiments and LC3B-I and PINK1 expression were measured by western blot analysis. As shown in Fig. 4i, LC3B-I was significantly reduced in the PX12+BTZ treatment group. Furthermore, PINK1 expression was significantly increased in PX12+BTZ treatment group. These data demonstrated the activation of mitophagy in vivo with PX12+BTZ treatment, which was consistent with our in vitro findings.

To determine if mitophagy activation mediates the effects of PX12+BTZ combination in BTZ-resistant multiple myeloma cells, we used bafilomycin, a mitophagy inhibitor, to block mitophagy. MM.1R-BTZ myeloma cells and RPMI8226/Dox-BTZ myeloma cells were treated with PX12, BTZ, or PX12+BTZ with or without 5 μM of bafilomycin. As shown in Fig. 4j, k, bafilomycin reversed the inhibitory effect mediated by PX12+BTZ treatment. Taken together, our results supported our hypothesis that thioredoxin inhibition enhances bortezomib sensitivity in BTZ-resistant myeloma cells via mitophagy activation.

**Fig. 5:**
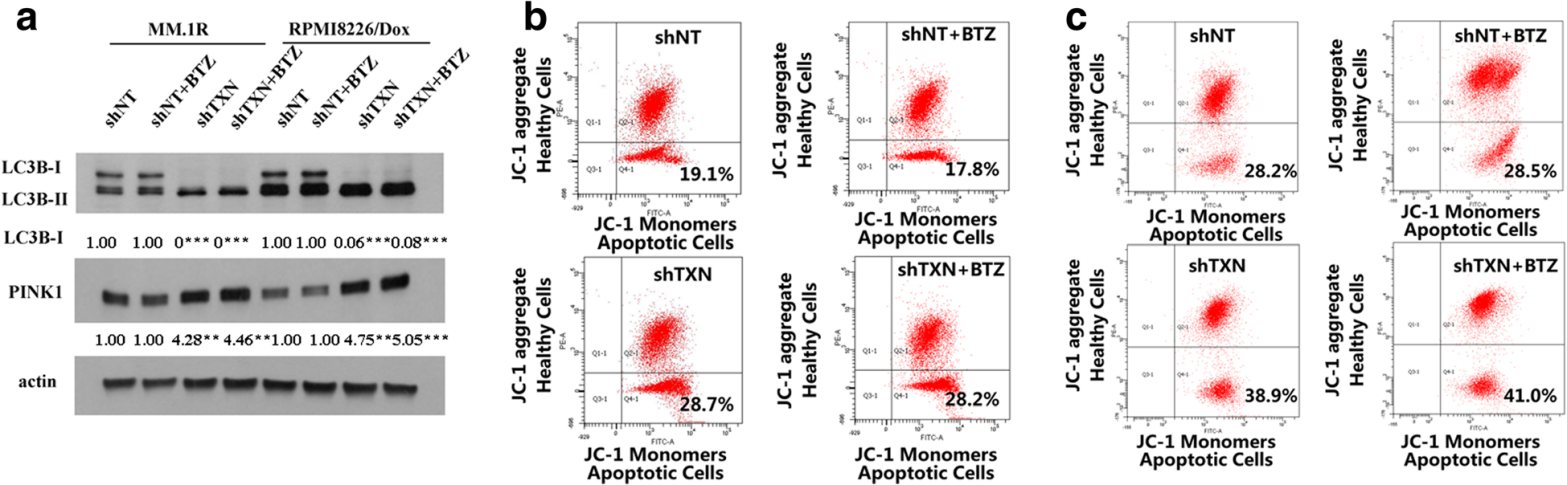
Genetic deletion of thioredoxin using shRNA triggers mitophagy in BTZ-resistant multiple myeloma cells. **a** Western blot analyses of LC3B and PINK1 expression in MM.1R- and RPMI8226/Dox-BTZ-resistant cells stably transfected with shNT and shTNX. **b** JC-1 fluorescence intensity in MM.1R-BTZ-resistant cells stably transfected with shNT and shTNX, and treated with or without BTZ (5 nM). **c** JC-1 fluorescence intensity in RPMI8226/Dox-BTZ-resistant cells stably transfected with shNT and shTNX and treated with or without BTZ (5 nM). The intensity of expression was semi-quantitated using Image-Pro Plus 6.0 software and adjusted to β-actin. Error bars, standard error of the mean (SEM); *p < 0.05, **p < 0.01, ***p < 0.005, ****p < 0.001

Similarly, genetic deletion of *TXN* in MM.1R- and RPMI8226/Dox-BTZ myeloma cells resulted in increased expression of PINK1 and LC3B-II, and the mitochondrial membrane depolarization as measured by JC-1 aggregates (Fig. 5), consistent with the induction of mitophagy.

### Inhibition of thioredoxin decreases mTOR and ERK1/2 phosphorylation

**Fig. 6:**
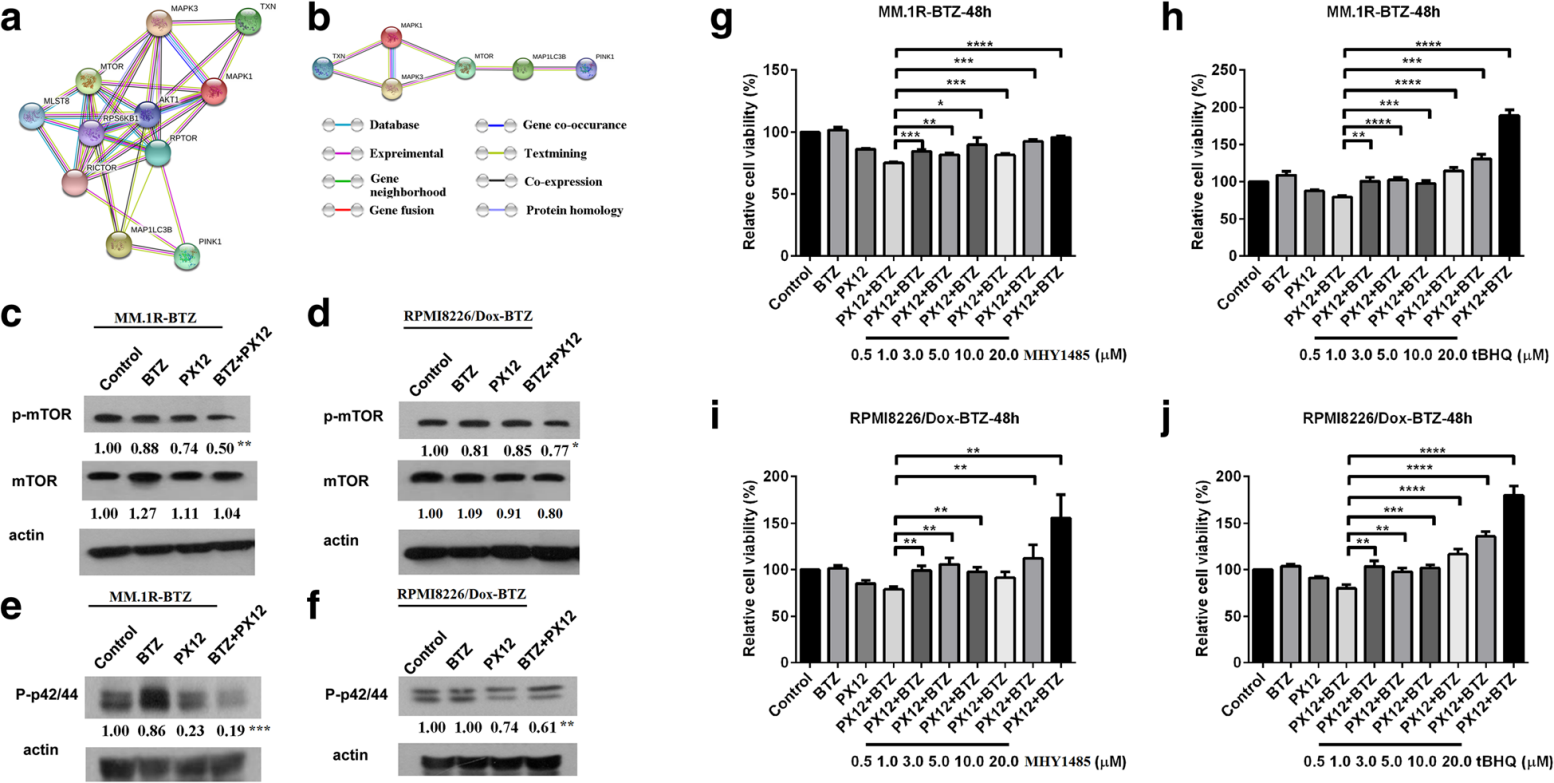
Inhibition of thioredoxin decreases mTOR and ERK1/2 phosphorylation. **a** Genetic interaction network associated with mitophagy and thioredoxin. **b** Optimized network associated with mitophagy and thioredoxin. In this network, circle represents node (proteins), while line represents edge (connections). **c** Western blot analysis of mTOR and p-mTOR expression in MM.1R-BTZ-resistant cells treated with BTZ (5 nM) and/or PX12 (2.5 μM) treatment for 48 h. **d** Western blot analysis of mTOR and p-mTOR expression in RPMI8226/Dox-BTZ-resistant cells treated with BTZ (5 nM) and/or PX12 (2.5 μM) treatment for 48 h. **e** Western blot analysis of p-ERK1/2 expression in MM.1R-BTZ-resistant cells treated with BTZ (5 nM) and/or PX12 (2.5 μM) for 48 h. **f** Western blot analysis of p-ERK1/2 expression in RPMI8226/Dox-BTZ-resistant cells treated with BTZ (5 nM) and/or PX12 (2.5 μM) treatment for 48 h. The intensity of expression was semi-quantitated using Image-Pro Plus 6.0 software and adjusted to β-actin. **g** Cell viability of MM.1R-BTZ-resistant cells treated with BTZ (5 nM) or/and PX12 (2.5 μM) with or without mTOR activator MHY1485 (range from 0.5 to 20 μM). **h** Cell viability of MM.1R-BTZ-resistant cells treated with BTZ (5 nM) or/and PX12 (2.5 μM) with or without ERK activator tBHQ (range from 0.5 to 20 μM). **i** Cell viability of RPMI8226/Dox-BTZ-resistant cells treated with BTZ (5 nM) or/and PX12 (2.5 μM) with or without mTOR activator MHY1485 (range from 0.5 to 20 μM). **j** Cell viability of RPMI8226/Dox-BTZ-resistant cells treated with BTZ (5 nM) or/and PX12 (2.5 μM) with or without ERK activator tBHQ (range from 0.5 to 20 μM). Each experiments were repeated three times. Error bars, standard error of the mean (SEM); *p < 0.05, **p < 0.01, ***p < 0.005, ****p < 0.001

To explore the molecular mechanisms by which thioredoxin inhibition leads to the activation of mitophagy and cytotoxicities in BTZ-resistant multiple myeloma cells, we established an in silico genetic interaction network-predicted method using String (https://string-db.org/). This network analysis predicts several key hubs associated with mitophagy and thioredoxin according to the literatures search. Figure 6a, b revealed the predicted key proteins including mTOR, ERK1/2, and PINK1 and their potential relationships with mitophagy. We subsequently performed western blot analysis to validate these interactions. Our results indicated that although the total protein levels were not affected, the phosphorylation level of mTOR (Fig. 6c, d) and ERK1/2 (Fig. 6e, f) decreased dramatically in MM.1R-BTZ- and RPMI8226/Dox-BTZ-resistant cells treated with BTZ+PX12, when compared to those in the BTZ group.

To better understand whether the reduced levels of mTOR and ERK1/2 phosphorylation are directly involved in the anti-myeloma effects of thioredoxin inhibition in bortezomib-resistant myeloma cells, we treated MM.1R-BTZ and RPMI8226/Dox-BTZ-resistant cells with the BTZ+PX12 combination in the absence and presence of various concentration of mTOR activator (MHY1485) or ERK activator (tBHQ). As shown in Fig. 6g–j, the inhibitory effects of BTZ and PX12 co-treatment were reversed by MHY1485 or tBHQ in a dose-dependent manner. Thus, our data demonstrate that mTOR and ERK1/2 phosphorylation could play a role in the effects mediated by BTZ+PX12 treatment in bortezomib-resistant myeloma cells.

### High expression of *TXN* is associated with poor prognosis in multiple myeloma patients

**Fig. 7:**
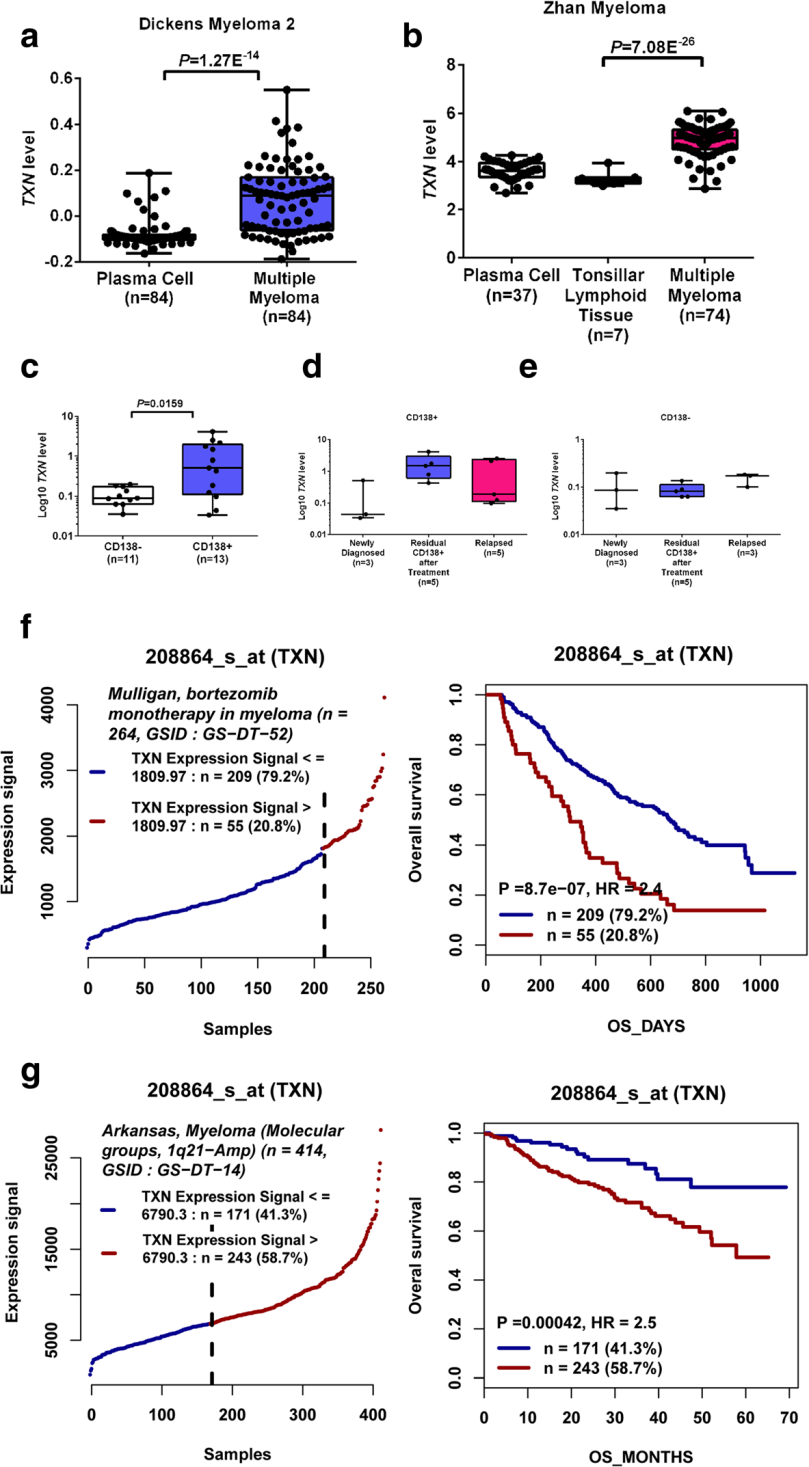
High Expression of *TXN* is associated with poor prognosis in patients with myeloma. **a**–**b** *TXN* expression levels in normal plasma cells and myeloma cells from Dickens and Zhan myeloma microarray datasets. **c** *TXN* expression in CD138^+^ and CD138^−^ cells from patients with multiple myeloma. **d** *TXN* expression in CD138^+^ samples from patients with newly diagnosed, treated, and relapsed myeloma. **e** *TXN* expression in CD138^−^ samples from patients with newly diagnosed, treated, and relapsed myeloma. Expression levels are presented as boxplots and were compared using an unpaired Student’s *t* test. **f**–**g** Kaplan-Meyer analysis of overall survival in Mulligan (**f**) and Arkansas (**g**) myeloma microarray. Survival analysis was performed using a log-rank test. *p < 0.05, **p < 0.01, ***p < 0.005, ****p < 0.001

To investigate the clinical significance of *TNX* in multiple myeloma, we interrogated publicly available microarray datasets and checked *TNX* mRNA level expression between normal plasma cells and multiple myeloma using the Oncomine database. As shown in Fig. 7, *TNX* expression was significantly increased in the CD138+ plasma cells of patients with myeloma compared to normal plasma cells and tonsillar lymphoid tissues in two different datasets [24, 25] (Fig. 7a, b).

To determine the expression of thioredoxin in bortezomib relapsed/refractory myeloma patients, we isolated CD138^+^ cells and CD138^−^ cells from untreated newly diagnosed myeloma patients (*n* = 3), myeloma patients who have just completed bortezomib containing regimen induction therapy with residual myeloma cells (*n* = 5), and relapsed/refractory myeloma patients (*n* = 5) who had previous exposure and resistant to bortezomib. When compared to the CD138^−^ cells, *TNX* mRNA was significantly increased in the CD138^+^ plasma cells, suggesting that TXN is upregulated in myeloma cells in comparison to non-myelomatous cells of the same patient (Fig. 7c). Furthermore, consistent with the findings in our adaptive BTZ-resistant myeloma cell lines, *TXN* expression demonstrated a trend for elevation in CD138^+^ myeloma cells after treatment with bortezomib or being refractory to bortezomib (Fig. 7d) *TNX* expression has no notable difference in CD138^−^ non-myelomatous cells among new diagnosed, treated, and refractory myeloma patients (Fig. 7e).

In addition, we analyzed the correlation between thioredoxin expression and overall survival using the Mulligan et al. [26] and Arkansas [27] microarray datasets. Compared to myeloma patients with low *TXN* expression, patients with higher *TXN* expression was associated with a shorter overall survival (Fig. 7f, g). Altogether, these findings demonstrated the clinical significance for *TXN* in myeloma, especially in bortezomib-resistant multiple myeloma.

## Discussion

Multiple myeloma remains an incurable disease and nearly all myeloma patients will eventually develop resistance to currently available therapeutic agents including proteasome inhibitors. Bortezomib is the first proteasome inhibitor used in the treatment of myeloma, and it has dramatically changed the landscape of the care of patients with multiple myeloma. Bortezomib can induce deeper response, lead to higher response rate, and extend the survival of patients with newly diagnosed multiple myeloma and of patients with relapsed myeloma. Unfortunately, development of drug resistance to bortezomib is inevitable and poses a major challenge in our continuous improvement of clinical outcomes for patients with multiple myeloma. Therefore, it is critical and imperative to elucidate the molecular mechanisms associated with or driving the development of bortezomib resistance. This information is essential in our efforts to overcome or re-sensitize bortezomib resistance.

In the current study, we generated over a period of 1.5 years several adaptive bortezomib-resistant myeloma cell lines (Fig. 1). We have also analyzed and compared the expression of thioredoxin in CD138^+^ myeloma cells from newly diagnosed myeloma patients, myeloma patients that were treated with bortezomib containing regimen, and relapsed/refractory myeloma patients who had prior exposure to bortezomib (Fig. 7). We have demonstrated that over-expression of thioredoxin was associated with the development of drug resistance to bortezomib. Thioredoxin was previously found to promote tumor growth through inhibition of apoptosis, reduce sensitivity of the tumor to drugs [28–30], and be associated with poor prognosis [29]. Raninga et al. has recently shown that compared to normal plasma cells, multiple myeloma cells had higher intrinsic oxidative stress and higher levels of thioredoxin and thioredoxin reductase expression and that thioredoxin over-expression was associated with resistance to NF-κβ inhibitors [28]. Using comparative proteomic profiling, Dytfeld et al. showed upregulation of thioredoxin expression in bortezomib-resistant myeloma cells [29]. Our current studies were consistent with and validated these observations. Importantly, our current studies advance significantly from those studies. We indeed showed that inhibition of thioredoxin by shRNA knockdown or pharmacological approach with PX12 could re-sensitize the bortezomib-resistant myeloma cells to bortezomib in vitro and in vivo in myeloma xenograft mouse models (Figs. 2 and 3). Mechanistically, we have demonstrated that inhibition of thioredoxin re-sensitize bortezomib-resistant myeloma cells through the activation of mitophagy. Our studies provide molecular rationale and justification for targeting thioredoxin in the treatment of bortezomib relapsed/refractory myeloma.

We found that PX12-sensitized BTZ-resistant myeloma cells to bortezomib through the activation of mitophagy. We confirmed the activation of mitophagy using several methods including LC3 western blotting, PINK1 expression analysis, measurement of mitochondrial membrane potential, and the visualization of mitochondrial-autophagosome fusion with transmission electron microscopy (Fig. 4). Using bafilomycin to inhibit mitophagy, we have demonstrated the important role of mitophagy in PX12-mediated effects. Thioredoxin-specific shRNA knockdown revealed similar findings on the induction of mitophagy (Fig. 5). Thioredoxin and thioredoxin system maintain the intracellular redox homeostasis by scavenging ROS and regulating other redox proteins [31, 32]. Inhibition of thioredoxin increases intracellular oxidative stress [33, 34]. Increased production of ROS stimulates the initiation of mitophagy [28]. Mitophagy is a cellular self-cannibalization process that captures and digests mitochondrial in lysosomes [35]. The role of mitophagy in tumorigenesis remains a topic of debate. On one hand, mitophagy is activated in transformed cells and is beneficial for tumor maintenance and progression. On the other hand, excessive autophagy can act as a tumor-suppressive mechanism possibly through initiation of cell death or senescence [36–40]. The outcome of mitophagy activation in cancer depends on the stage of the disease, cell types, oncogenic drivers, and the intensity of the activation signal [35, 41, 42]. Mitophagy is also found to be involved in chemoresistance. Our current study suggested that reduced level of mitophagy is associated with the development of drug resistance in multiple myeloma cells and that activation of mitophagy could re-sensitize myeloma cells to bortezomib killing. Our study provides evidence for future exploring mitophagy pathway for the treatment of relapsed and refractory multiple myeloma.

In the current study, we delineated the molecular events involved in the PX12 + BTZ- induced mitophagy in MM cells. We described 2 signal proteins (p-mTOR and p-ERK1/2) that may play a role in PX12 + BTZ -induced mitophagy and anti-myeloma effects in MM cells. Using chemical activator of mTOR and ERK, we demonstrated an important role of mTOR and ERK in the anti-myeloma effects induced by thioredoxin inhibition. Previous studies have demonstrated that various signal pathways are involved in autophagy, including PI3K/Akt/mTOR, ERK1/2 and NF-*κ*B. mTOR (mechanistic target of rapamycin kinase) has been shown to control multiple cellular functions such as gene transcription, protein formation, cell proliferation and senescence and cell metabolism [43]. mTOR pathway is the master regulator of cellular metabolism and has been shown to be the major pathway regulating mitophagy. AKT was previously reported to control mitochondrial biogenesis and autophagy. In our system, we did not observe changes in AKT expression or phosphorylation after PX12 treatment. In addition, we found that treatment with PX12 resulted in desphosphorylation of ERK1/2. Our study suggested that inhibition of thioredoxin affects several molecular pathways that are important in the regulation of mitophagy.

ERK1 (p44) and ERK2 (p42) are two isoforms of ERK that are activated downstream of Ras in response to extracellular cues. ERK has been shown to induce autophagy in response to a number of anti-tumor/cytotoxic agents [44, 45]. Inhibition of ERK was associated with a decrease in autophagy and increased cellular sensitivity to tumor necrosis factor-α (TNF) in breast cancer MCF-7 cells [46]. Interestingly, our data showed that dephosphorylation of ERK1/2 appears to be associated with the induction of mitophagy in BTZ-resistant MM cells. Additional studies are needed to further understand the ERK1/2 pathway in BTZ resistance in multiple myeloma. The discrepancy between reduced activity of ERK1/2 in our current study and the findings in breast cancer could be due to different cancer type or to different drug sensitivity.

## Conclusions

In summary, we showed that upregulation of thioredoxin expression correlated with the development of bortezomib drug resistance in multiple myeloma. Inhibition of thioredoxin by shRNA or thioredoxin inhibitor re-sensitized bortezomib-resistant myeloma cells to bortezomib treatment. We further showed that the combination of bortezomib and thioredoxin inhibitor suppressed the growth of bortezomib-resistant myeloma cells through mitophagy activation. Our study provides new insights in the molecular mechanisms associated with bortezomib drug resistance. Our work was the first to demonstrate the novel role of mitophagy in multiple myeloma treatment.

## Additional file

Additional file 1: **Figure S1.** Effect of BTZ on cell survival and thioredoxin expression on the parental and resistant multiple myeloma cell lines following 48 h of BTZ treatment in vitro. (A–C) Generation of adaptive bortezomib-resistant myeloma cell lines: MM.1S cells (A), OPM1 cells (B), and NCIH929 (C) were cultured with serially increased concentrations of bortezomib over a period of 1.5 years. The parental and bortezomib-resistant myeloma cells were treated with various concentration of bortezomib for 48 h and the IC_50_ for parental and bortezomib-resistant myeloma cells were calculated and shown. (D–F) Thioredoxin mRNA expression is upregulated in bortezomib (BTZ)-resistant MM.1S (D), OPM1 (E), and NCIH929 (F) cells. Parental cells and BTZ-resistant MM.1S, OPM1, and NCIH929-resistant RPMI8226/Dox cells at the highest bortezomib concentration were harvested and mRNA expression measured by RT-PCR. Error bars, standard error of the mean (SEM). (G–I) Thioredoxin protein expression increased in parallel to increased bortezomib drug resistance. Serial MM.1S (G), OPM1 (H) and NCIH929 (I) cells that were cultured with increased concentrations of bortezomib were harvested and protein expression measured by western blot analysis. The intensity of expression was semi-quantitated using Image-Pro Plus 6.0 software and adjusted to β-actin. Error bars, standard error of the mean (SEM); *p < 0.05, **p < 0.01, ***p < 0.005, ****p < 0.001. (TIFF 239 kb)

## Abbreviations

BTZ: Bortezomib
ERK1/2: Extracellular signal-regulated protein kinases 1 and 2
MM: Multiple myeloma
mTOR: Mechanistic target of rapamycin kinase
PINK1: PTEN-induced putative kinase 1
TNF: Tumor necrosis factor-α
