# Supplementary figures and images for: Inhibition of thioredoxin activates mitophagy and overcomes adaptive bortezomib resistance in multiple myeloma
Source: J Hematol Oncol. 2018 Feb 27;11:29. doi: 10.1186/s13045-018-0575-7 (PMC5828316; doi:10.1186/s13045-018-0575-7)

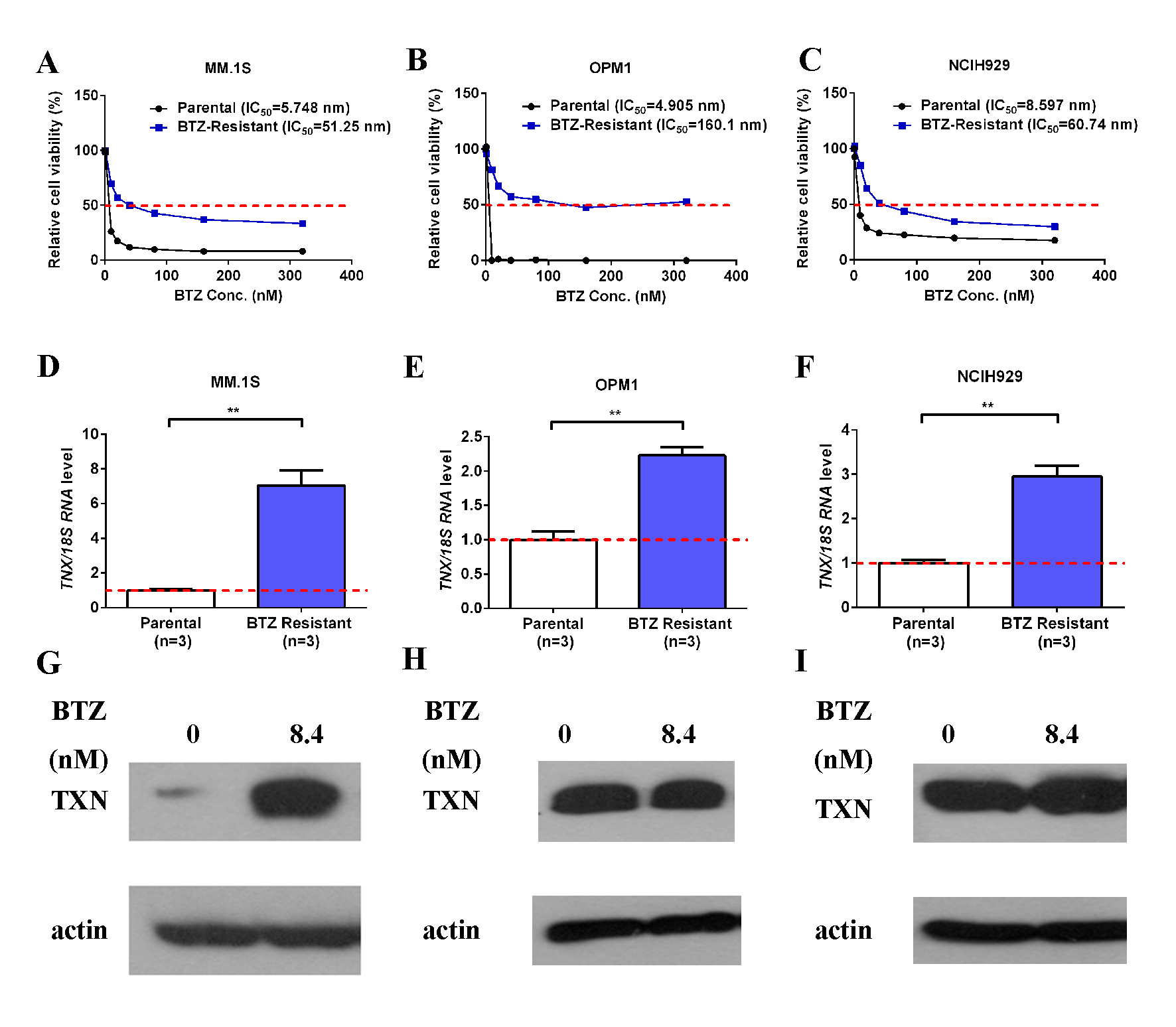

Supplement: Supplementary file 1 — Figure S1. Effect of BTZ on cell survival and thioredoxin expression on the parental and resistant multiple myeloma cell lines following 48 h of BTZ treatment in vitro. (A–C) Generation of adaptive bortezomib-resistant myeloma cell lines: MM.1S cells (A), OPM1 cells (B), and NCIH929 (C) were cultured with serially increased concentrations of bortezomib over a period of 1.5 years. The parental and bortezomib-resistant myeloma cells were treated with various concentration of bortezomib for 48 h and the IC50 for parental and bortezomib-resistant myeloma cells were calculated and shown. (D–F) Thioredoxin mRNA expression is upregulated in bortezomib (BTZ)-resistant MM.1S (D), OPM1 (E), and NCIH929 (F) cells. Parental cells and BTZ-resistant MM.1S, OPM1, and NCIH929-resistant RPMI8226/Dox cells at the highest bortezomib concentration were harvested and mRNA expression measured by RT-PCR. Error bars, standard error of the mean (SEM). (G–I) Thioredoxin protein expression increased in parallel to increased bortezomib drug resistance. Serial MM.1S (G), OPM1 (H) and NCIH929 (I) cells that were cultured with increased concentrations of bortezomib were harvested and protein expression measured by western blot analysis. The intensity of expression was semi-quantitated using Image-Pro Plus 6.0 software and adjusted to β-actin. Error bars, standard error of the mean (SEM); *p < 0.05, **p < 0.01, ***p < 0.005, ****p < 0.001. (TIFF 239 kb) [file 13045_2018_575_MOESM1_ESM.tif]
